# Biophysical characterization of Atg11, a scaffold protein essential for selective autophagy in yeast

**DOI:** 10.1002/2211-5463.12355

**Published:** 2017-12-04

**Authors:** Hironori Suzuki, Nobuo N. Noda

**Affiliations:** ^1^ Institute of Microbial Chemistry (BIKAKEN) Tokyo Japan

**Keywords:** Atg11, coiled‐coil, preautophagosomal structure, scaffold protein, selective autophagy

## Abstract

Autophagy is an intracellular degradation system in which the formation of an autophagosome is a key event. In budding yeast, autophagosomes are generated from the preautophagosomal structure (PAS), in which Atg11 and Atg17 function as scaffolds essential for selective and nonselective types of autophagy, respectively. Structural studies have been extensively performed on Atg17, but not on Atg11, preventing us from understanding the selective type of the PAS. Here, we purified and characterized Atg11. Biophysical analyses, including analytical ultracentrifugation and CD, showed that Atg11 behaves as an elongated homodimer abundant in α‐helices in solution. Moreover, truncation analyses suggested that Atg11 has a parallel coiled‐coil architecture, in contrast to the antiparallel dimeric architecture of Atg17.

**Abbreviations**
Atg11_C: C‐terminal region of Atg11
Atg11_N: N‐terminal region of Atg11
Atg: autophagy‐related
MBP: maltose‐binding protein
PAS: preautophagosomal structure

Autophagy is an intracellular degradation system widely conserved in eukaryotes, including yeasts and mammals 1. When autophagy is induced, for example, upon starvation, a double‐membrane structure called an autophagosome is generated, which isolates a part of the cytoplasm including proteins and organelles. Autophagosomes then fuse with the vacuole in yeast and plants or with the lysosomes in mammals, and the isolated contents are degraded by vacuolar/lysosomal hydrolases. Autophagy mediates renovation of intracellular components by nonselective degradation 2. In addition, autophagy also mediates selective degradation of protein aggregates, damaged mitochondria, a portion of endoplasmic reticulum and nucleus, and even bacteria that invade cells, a process referred to as selective autophagy 3, 4, 5, 6. Selective autophagy is believed to be involved in the prevention of various severe diseases, including cancer, neurodegeneration, and infections 2, 7. In spite of its medical importance, the fundamental molecular mechanisms underlying selective autophagy have remained largely unknown.

In the budding yeast *Saccharomyces cerevisiae*, ~ 40 autophagy‐related (Atg) proteins that regulate autophagy have been identified 1. Of them, 18 Atg proteins, referred to as core Atgs, are essential for autophagosome formation during starvation‐induced, nonselective autophagy. These core Atgs colocalize to the preautophagosomal structure (PAS), from which autophagosomes are believed to be generated 8. The scaffold protein Atg17 forms a higher‐order assemblage with the help of the intrinsically disordered protein Atg13 and plays an essential role in organizing starvation‐induced PAS 9, 10. In selective autophagy, Atg11 is known to play an essential role as a scaffold protein instead of Atg17 and is thought to play a key role in organizing selective autophagy‐specific PAS (selective PAS) via interacting with various Atg proteins (Fig. 1A) 11, 12, 13. In addition, formation of the selective PAS requires selective cargo itself and cargo‐specific receptor proteins such as Atg19 14. Therefore, the Atg11–receptor–cargo complex is thought to organize the selective PAS.

**Figure 1:**
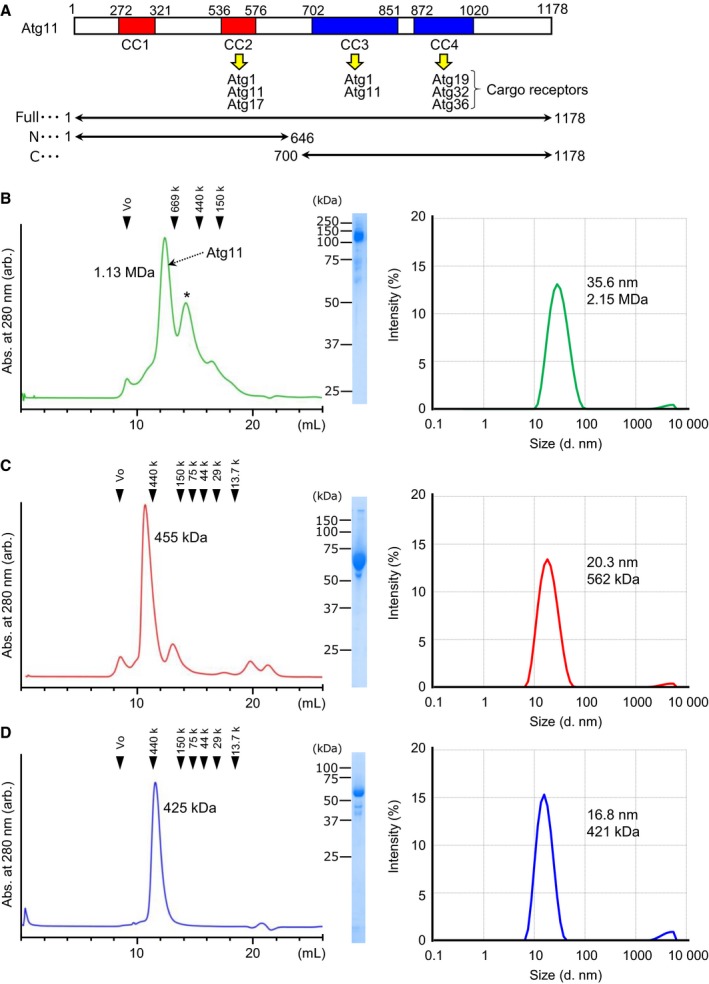
Atg11 behaves as a monodispersed large particle in solution. (A) Summary of the known functional regions of Atg11. CC1–4 indicates predicted coiled‐coils, and each binding partner is listed. The constructs used in this study are shown below. (B–D) Elution profiles of the size exclusion chromatography are shown left, the SDS/PAGE pattern of the peak fraction is shown in the middle, and the size distribution of Atg11 measured by DLS is shown on the right. (B) Full‐length Atg11, (C) Atg11_N, and (D) Atg11_C. An asterisk indicates nonspecific or degraded proteins.

Recently, structural biological studies have been extensively performed on Atg17 and Atg13 and have revealed the architecture of starvation‐induced PAS and the molecular mechanisms of the initiation of starvation‐induced autophagy 9, 10, 15, 16, 17, 18. In contrast, no structural studies have been performed on Atg11. In order to elucidate the molecular mechanisms of selective autophagy, it is essential to determine the structure of Atg11, which functions as the principal component in selective PAS. Here, we report the purification and characterization of Atg11 using biophysical methods, which revealed that Atg11 behaves as a homodimer with an elongated α‐helical conformation in solution. Moreover, truncation analysis showed that both terminal halves of Atg11 form a dimer, suggesting that Atg11 has a parallel coiled‐coil architecture that is distinct from Atg17.

## Materials and methods

### Expression and purification of Atg11 proteins

The genes encoding full‐length *S. cerevisiae* Atg11 (residues: 1–1178; Atg11), the N‐terminal region (residues: 1–646; Atg11_N), and the C‐terminal region (residues; 700–1178; Atg11_C) were amplified by PCR using PrimeSTAR Max polymerase (Takara, Shiga, Japan). The full‐length Atg11 product was subcloned into the downstream region of the PH promoter of modified insect cell expression vector pFastBac Dual, whereas those for Atg11_N and Atg11_C were subcloned into pET‐23a and a modified pMAL‐c2E vector encoding maltose‐binding protein (MBP) following the HRV3C protease site, respectively. Full‐length Atg11 whose C terminus was attached with a HRV3C protease site followed by FLAG and 6× His tags was expressed in High Five insect cells using the baculovirus expression system (Life Technologies, Gaithersburg, MD, USA). After viral infection, the cells were cultured at 27 °C for 48 h. Cells were harvested by centrifugation, resuspended in buffer containing 50 mm Tris pH 8.0, 500 mm NaCl, 10 mm imidazole, 10% glycerol, 2 mm MgCl_2_, 10 U·mL^−1^ benzonase (Merck, Darmstadt, Germany), and 0.2 mm phenylmethylsulfonyl fluoride, and lysed by sonication. After centrifugation, the supernatant containing the target proteins was loaded onto a Talon metal affinity resin column (Clontech, Palo Alto, CA, USA) and eluted with 50 mm Tris pH 8.0, 100 mm NaCl, and 100 mm imidazole. The eluted proteins were further purified using a HiTrap Q anion exchange column. The His‐tag was then cleaved using HRV3C protease overnight at 4 °C. After removal of the HRV3C protease using GST accept resin (Nacalai Tesque Inc., Kyoto, Japan) and cleaved His‐tag and contaminated proteins using Talon metal affinity resin, the proteins were finally purified using a Superose6 Increase size exclusion column (GE Healthcare, Piscataway, NJ, USA) in 20 mm Tris pH 8.0, 300 mm NaCl, and 1 mm DTT.

The plasmids of Atg11_N and Atg11_C were transformed into *Escherichia coli* BL21(DE3) cells. The cells were cultured at 37 °C until the optical density at 600 nm reached 0.6. Isopropyl β‐d‐1‐thiogalactopyranoside was then added to a final concentration of 0.1 mm, and the cells were further cultured at 18 °C overnight. The cells were collected by centrifugation and disrupted by sonication in 50 mm Tris pH 8.0 and 500 mm NaCl (for Atg11_N, 10 mm imidazole was added). 6× His‐tagged Atg11_N was initially purified using a Talon metal affinity column, followed by tandem chromatography using a HiTrap Q anion exchange column and a HiLoad 26/60 Superdex200 size exclusion column (GE Healthcare). MBP‐fused Atg11_C was initially purified using an Amylose column (New England BioLabs, Beverly, MA, USA). The MBP tag was then cleaved with HRV3C protease overnight at 4 °C. After removing HRV3C protease using GST accept resin (Nacalai Tesque), Atg11_C was further purified by tandem chromatography using an SP‐Sepharose cation exchange column (GE Healthcare) and a HiLoad 26/60 Superdex 200 size exclusion column.

### Gel filtration analysis

Purified Atg11 was applied to a Superose 6 Increase 10/300 GL column in 20 mm Tris pH 8.0, 300 mm NaCl, and 1 mm DTT, whereas purified Atg11_N and Atg11_C were applied to a Superdex 200 Increase 10/300 GL column in 20 mm Tris pH 8.0 and 150 mm NaCl. The Superose 6 Increase 10/300 GL column was calibrated with blue dextran (2000 kDa), thyroglobulin (669 kDa), apoferritin (440 kDa), and alcohol dehydrogenase (150 kDa), purchased from Merck. The Superdex 200 Increase 10/300 GL column was calibrated with apoferritin (440 kDa), alcohol dehydrogenase (150 kDa), conalbumin (75 kDa), ovalbumin (44 kDa), carbonic anhydrase (29 kDa), and ribonuclease (13.7 kDa), purchased from GE Healthcare. The eluted proteins were detected by absorbance at 280 nm.

### CD spectroscopy

CD spectra were recorded at 20 °C on a Jasco J‐720W spectropolarimeter equipped with a Julabo F25‐ED temperature controller. CD spectra were collected over the wavelength range 195–260 nm at a resolution of 0.1 nm, a bandwidth of 1 nm, and a response time of 1 s. Final spectra were the sum of 16 scans accumulated at a speed of 50 nm·min^−1^. Proteins used for CD measurement were prepared at the concentration of 0.1 mg·mL^−1^ in the buffer consisting of 2 mm Tris/HCl pH 8.0, 150 mm NaCl.

### Dynamic light scattering measurement

Proteins used for DLS measurement were prepared at the concentration of 1 mg·mL^−1^ in the buffer consisting of 20 mm Tris/HCl pH 8.0, 150 mm NaCl for Atg11_N and Atg11_C and that consisting of 20 mm Tris/HCl pH 8.0, 300 mm NaCl, 1 mm DTT for full‐length Atg11. Proteins were centrifuged at 15 000 ***g*** for 15 min before measurement. The samples were loaded into a quartz batch cuvette ZEN2112 (Malvern, Worcestershire, UK), and three continuous measurements were taken at 25 °C using a Zetasizer Nano S system (Malvern). The averages of three measurements are shown in Fig 1B–D.

### Analytical ultracentrifugation

**Figure 2:**
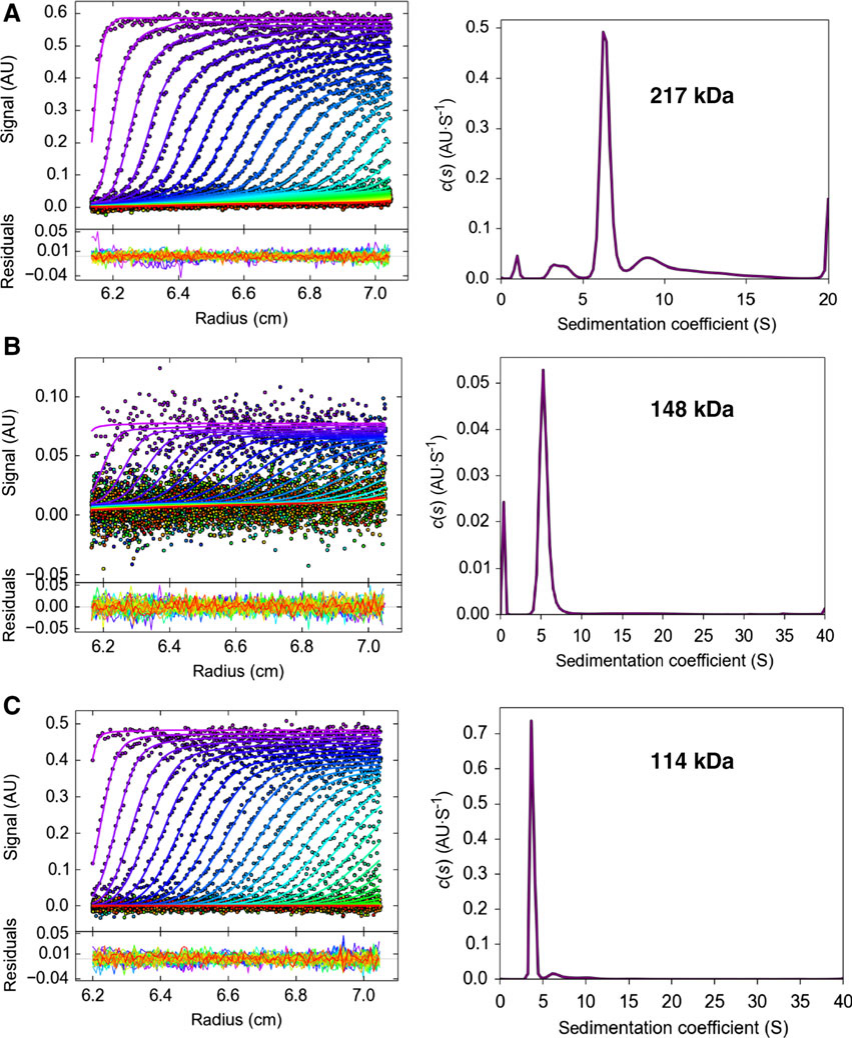
Dimer formation of Atg11 (A), Atg11_N (B), and Atg11_C (C), as revealed by analytical ultracentrifugation. The upper panels show the distribution of sedimentation coefficient, c(s), while the lower panels show the development of the moving boundaries superimposed on those calculated from the c(s) values.

Sedimentation velocity experiments were performed on an Optima XL‐I ultracentrifuge (Beckman Coulter, Brea, CA, USA) using an An50Ti rotor at 36 000 r.p.m. (Atg11_N and Atg11_C) or 42 000 r.p.m. (full‐length Atg11) at 20 °C (Fig 2). Charcoal‐filled Epon double‐sector cells were used. Full‐length Atg11 at the concentration of 0.65 mg·mL^−1^ in 20 mm Tris pH 8.0, 300 mm NaCl and 1 mm DTT, Atg11_N at the concentration of 0.61 mg·mL^−1^ in 20 mm Tris pH 8.0, 150 mm NaCl, and Atg11_C at the concentration of 0.92 mg·mL^−1^ in the same buffer as Atg11_N were used for measurement. The same buffers for each protein were used in the reference sectors. The sedimentation coefficient distribution function, c(s), was obtained using the sedfit program 19, 20. Partial specific volumes of proteins were calculated based on their amino acid sequences using the Sednterp program 21. The solvent density and viscosity were also calculated using this program. Molecular weights were obtained by converting c(s) to c(M) with SEDFIT based on the Svedberg equation using the obtained sedimentation coefficients and the diffusion coefficients.

## Results

Atg11 is the second largest protein (1178 amino acids, molecular weight 135 kDa) when compared with other core Atg proteins and is difficult to purify, which has impeded structural studies despite its immense significance in selective autophagy. We first tried to express full‐length Atg11 using *E. coli*, but failed to obtain soluble proteins. Therefore, we next used insect cells with the baculovirus expression system and were able to produce sufficient amounts of soluble Atg11. Through five consecutive steps of chromatography, we purified Atg11 to near homogeneity (Fig. 1B). During purification, we noticed that some portions of Atg11 were cleaved into two, and the secondary structure prediction suggested that residues 647–699 of Atg11 lack secondary structures. We speculated that the region is susceptible to proteolysis and therefore designed two shortened versions of Atg11: Atg11_N (residues 1–646; molecular weight 75 kDa) and Atg11_C (700–1178; molecular weight 55 kDa), and succeeded in purifying both proteins from *E. coli*. Previous study suggests that both Atg11_N and Atg11_C are essential for selective PAS organization 13.

Using the purified proteins, we first performed size exclusion chromatography (Fig. 1B–D, left panels). Full‐length Atg11 eluted as a large protein with an estimated molecular weight of 1.13 MDa, which corresponds to 8–9 copies of Atg11 (Fig. 1B, left). The elution profiles of Atg11_N and Atg11_C indicated molecular masses of 455 and 425 kDa, corresponding to 6 and 7–8 copies of each protein, respectively (Fig. 1C,D, left). These data suggested two possibilities: One possibility is that Atg11 behaves as an oligomer in solution and that both N‐ and C‐terminal halves of Atg11 can oligomerize independently with each other. Another possibility is that Atg11 is not so oligomerized but has a considerably elongated conformation. In order to study whether the peak fraction is monodispersed or not, and to estimate the particle size of the proteins, we next performed DLS measurements (Fig. 1B–D, right). The DLS data suggested that full‐length Atg11, Atg11_N, and Atg11_C all exist as monodispersed particle forms in solution and that each particle size has an estimated diameter of 35.6 nm, 20.3 nm, and 16.8 nm, which correspond to a molecular weight of 2.15 MDa, 562 kDa, and 421 kDa, respectively, when the particles are hypothesized to be globular.

Both the gel filtration and DLS data suggested that Atg11, Atg11_N, and Atg11_C exist as oligomers whose sizes are much larger than those of each monomer. However, both methods estimate the size by assuming that the proteins are globular and tend to give larger values than the actual weight when the shape of the protein is not globular. In order to determine the precise molecular weight and assembled number of Atg11 proteins in solution, we employed analytical ultracentrifugation, which estimates a molecular weight of a particle in solution independent of the molecular shape. Sedimentation velocity experiments indicated that Atg11, Atg11_N, and Atg11_C all behaved mainly as single particles with average sedimentation coefficients of 6.4, 5.4, and 3.8 S, respectively (Fig. 2). Using these values, the average molecular weight of Atg11, Atg11_N, and Atg11_C could be estimated to be 217 kDa, 148 kDa, and 114 kDa, which correspond to 1.6, 2.0, and 2.1 copies of each protein, respectively. These data suggest that Atg11, Atg11_N, and Atg11_C all exist as a homodimer in solution. Sedimentation velocity experiments also give us the values of the friction ratio, which approximates to 1.0 when the particle is perfectly spherical and becomes larger in proportion to the deviation of the shape from the globular form. The friction ratio of Atg11, Atg11_N, and Atg11_C was 1.86, 1.73, and 2.11, respectively, indicating that their shape is far from globular and largely extended and that Atg11_C is more elongated than Atg11_N.

**Figure 3:**
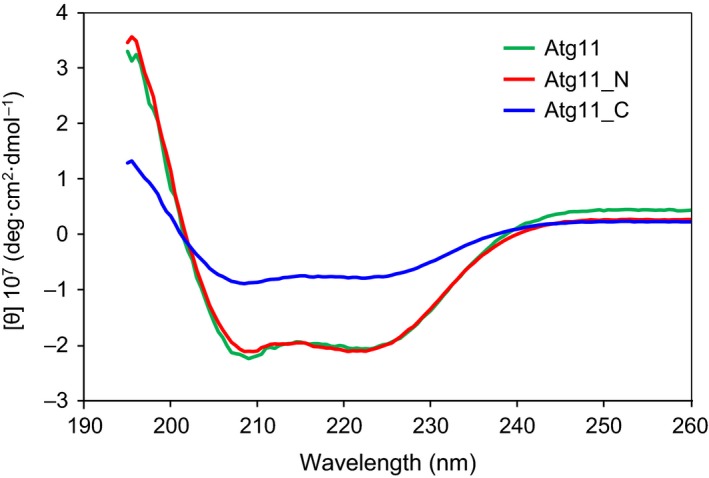
CD spectra of Atg11, Atg11_N, and Atg11_C.

Elongated dimeric proteins tend to be abundant in α‐helices, which frequently form superhelical structures called coiled‐coils 22, 23. As Atg11 is proposed to be elongated and dimeric, it can be speculated that Atg11 is abundant in α‐helices and has a dimeric coiled‐coil architecture. In fact, secondary structure predictions of Atg11 from its sequence suggested that Atg11 contains four coiled‐coil regions 13. In order to investigate the secondary structure composition of Atg11 in solution, we measured CD spectra of Atg11, Atg11_N, and Atg11_C, which showed similar patterns to each other, with two minimum peaks around 208 nm and 222 nm (Fig 3). These data indicated that these three proteins are abundant in α‐helices. Altogether, these data strongly suggest that Atg11 has an elongated, dimeric coiled‐coil architecture in solution.

## Discussion

In this report, we purified and characterized Atg11, a key scaffold protein in selective autophagy, using four different methods, and revealed that Atg11 behaves as an elongated homodimer abundant in α‐helices in solution. Coiled‐coils are the most common structural motif for constructing such architectures and occur widely in proteins involved in membrane dynamics, including autophagy. Of the 18 core Atg proteins, four (Atg6, Atg14, Atg16, and Atg17) are shown to possess coiled‐coils, which are involved in the formation of homodimers in the case of Atg6, Atg16, and Atg17, or heterodimers (Atg6–Atg14) 15, 24, 25, 26. Moreover, coiled‐coils were predicted to be abundant in Atg11 from its sequence 13. These observations suggest that the elongated dimeric architecture of Atg11 consists of coiled‐coils.

**Figure 4:**
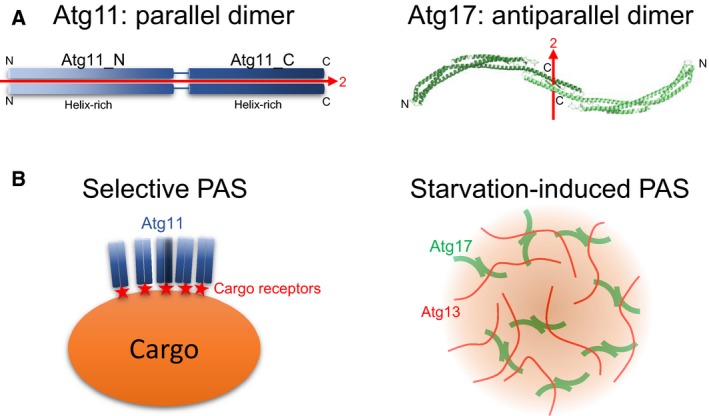
Proposed Atg11 architecture and model of selective PAS organization. (A) Left, schematic drawing of the Atg11 dimer. Right, crystal structure of the Atg17 dimer (PDB ID 5JHF). N and C indicate the N and C termini of each protomer, respectively. The pseudo‐two fold symmetrical axis is shown with a red arrow. (B) Proposed model of selective PAS (left) and starvation‐induced PAS (right). For simplicity, other factors involved in PAS have been omitted.

The dimerization mode induced by coiled‐coils can be classified into two: parallel and antiparallel, where two α‐helices are in the same or opposite amino‐to‐carboxy‐terminal direction, respectively 23. Parallel, but not antiparallel, coiled‐coils tend to retain a dimeric architecture after truncation. In the case of Atg11, both Atg11_N and Atg11_C behaved as stable homodimers, suggesting that the dimeric nature of Atg11 coiled‐coils is more likely to be parallel, as shown in Fig. 4A, left. This is in contrast to the antiparallel dimeric structure of Atg17 (Fig. 4A, right), a scaffold protein involved in nonselective autophagy, which was shown to disassemble into monomers upon truncation 15. The greatest structural difference between parallel and antiparallel coiled‐coils is that the former and the latter make a pseudo‐twofold symmetry whose axis is parallel or perpendicular to the coiled‐coil long axis, respectively (Fig. 4A). As a result, the overall structure of antiparallel, but not parallel, coiled‐coils becomes symmetrical and is suitable for functioning as a building block of higher‐order protein assemblage due to their ability to repeat protein–protein interactions in three‐dimensional directions. When Atg17 functions as a scaffold in starvation‐induced PAS, the intrinsically disordered protein Atg13 links symmetrical Atg17 dimers repeatedly, leading to the formation of a large assemblage (Fig. 4B, right) 10. Selective PAS requires a large cargo and receptors in addition to Atg11, all of which are dispensable in starvation‐induced PAS 14. In selective PAS, binding of multiple Atg11 dimers to the surface of the large cargo via receptors might be sufficient and would not require repeated interactions in three‐dimensional directions as is the case for Atg17 (Fig. 4B, left). Thus, the architectural difference between Atg11 and Atg17 reflects the difference in the PAS organization between selective and nonselective autophagy. In order to validate this hypothesis and to determine the architecture of selective PAS, high‐resolution structural studies on Atg11 are strongly required in the future.

## Author contributions

HS and NNN designed the experiments. HS performed all the experiments. HS and NNN analyzed the data and wrote the manuscript. NNN supervised the work.
